# Sheep and Goat Meat Processing and Quality

**DOI:** 10.3390/foods12102033

**Published:** 2023-05-17

**Authors:** Severiano R. Silva, Alfredo Teixeira, Cristina Guedes

**Affiliations:** 1 Veterinary and Animal Research Centre (CECAV) and Associate Laboratory of Animal and Veterinary Science (AL4AnimalS), University of Trás-os-Montes e Alto Douro, Quinta de Prados, 5000-801 Vila Real, Portugal; ssilva@utad.pt (S.R.S.); cguedes@utad.pt (C.G.); 2 Mountain Research Centre (CIMO) and Laboratory for Sustainability and Technology in Mountain Regions, Polytechnic Institut of Bragança, Campus de Santa Apolónia, 5300-253 Bragança, Portugal

Sheep and goat meat production includes the increased demand for grass-fed and organic meat and value-added products such as sausages, meatballs, and burgers. There has also been a growing worldwide interest in ethnic cuisine that uses sheep and goat meat. For example, Mediterranean and Middle Eastern dishes such as lamb kebabs and goat curries can be considered under this scope. There has also been greater interest in diversifying protein sources, including sheep and goat meat, as consumers seek better animal welfare and eco-friendly food choices. Several factors underpin these trends. The reasons for such trends are linked to the physicochemical, sensory, and nutritional properties and long tradition of consuming sheep and goat meat and meat products, such as consuming light carcasses of lambs and goat kids across EU Mediterranean countries, which are commercialized as quality meat brands with protected designation of origin or geographical indication provided. To evaluate and authenticate the origin and quality of sheep and goat meat and its products, non-invasive and non-destructive methods and techniques have gained relevance. These are topics that will be addressed in this Special Issue.

This Special Issue is made up of ten works—three reviews and seven research articles—written by researchers from diverse backgrounds that provide up-to-date research on aspects related to sheep and goat meat and meat products and also advances made in meat quality assessment. The different works can be assigned to three distinct groups. First, one group looking into the consumption of sheep and goat meat products around the world and strategies for producing healthier meat products from those species; another group, in which the effects of management practices, systems, and meat ageing time on the quality of sheep and goat meat are studied; and finally, a group in which techniques to evaluate and improve meat quality are examined.

Regarding sheep and goat meat products, Teixeira et al. [1] present a comprehensive overview of the origin and type of the most important sheep and goat processed meat products produced worldwide. The overview also highlights future trends in the production, processing, and commercial potential of sheep and goat processed meat products and their interest in food research. In this sense, the works of Sujarwanta et al. [2] and Teixeira et al. [3] introduce strategies for producing healthier goat meat products. In the first of these works, the substitution of tapioca for rice bran, which has a lower glycaemic index, was studied to create a healthier goat meatball product. It was concluded that substituting tapioca with up to 25% rice bran was deemed acceptable for Indonesian consumers. The latter work studied the effect of replacing pork as a source of fat with an olive oleogel in goat meat burgers, with the aim of modifying and improving the meat’s fatty acid profile. As a result, it was concluded that goat burgers had the highest MUFA and PUFA and better lipidic quality. Additionally related to the beneficial effects of such meat on human health, Ren et al. [4] studied the potential effect of using *Tamarix ramosissima* as barbecue skewers to reduce the formation of heterocyclic amines (HAs) in roast lamb meat. The results demonstrate that *Tamarix ramosissima* bark extract and the corresponding flavonoids inhibit the formation of 2-amino-1-methyl-6-phenylimidazo[4,5-*b*]pyridine, which is one of the most abundant HAs in food and may therefore have a beneficial effect on human health.

The influence of lamb meat ageing on the volatile compounds and sensory attributes of the meat was studied by Insauti et al. [5]. These authors analyzed the *Longissimus thoracis et lumborum* muscle of Navarra breed lambs. They concluded that the four-day ageing of lamb meat positively affects the meat’s sensory odour and flavor quality. On the other hand, Hoffman et al. [6] studied the effect of the production system (feedlot or free-range) used on the sensory quality characteristics of Dorper lambs. Feedlot lambs produced meat that was juicier and more tender than the meat from free-range lambs. However, free-range meat may not necessarily be distinguished from feedlot meat as far as aroma and flavor are concerned. In addition, in their work with lambs, Van der Merwe et al. [7] studied 148 carcasses of 7 breeds slaughtered at the same degree of fatness (Dohne Merino, Dormer, Dorper, Meatmaster, Merino, Namaqua Afrikaner, and South African Mutton Merino). Differences in carcass weight were observed, which explains the differences in maturity and the onset of fat deposition. For physical meat quality, relatively small differences were observed. For example, meat shear-force Dormer and Namaqua lambs showed higher values (~46 N) than Meatmaster carcasses (~34 N). Despite these differences, no influence on the perceived quality of the meat is expected.

In a study with suckling goat kids, Martinez et al. [8] studied the possibility that changes in sarcoplasmic and myofibrillar protein (MFP) fractions serve as welfare indicators in terms of farm management practices and transport time. For this particular study, 64 suckling goat kids from 2 farms with high and low welfare-friendly management practices and transported for 2 or 6 hours immediately before slaughter were used. In samples of *Longissimus thoracis et lumborum* ageing for 3, 8, and 21 days, muscle proteins were separated by electrophoresis in sodium dodecyl sulfate and polyacrylamide SDS-PAGE gel. Some MFP fractions from the *Longissimus lumborum* muscle were significantly influenced by poor management on the farm and can therefore be considered as welfare indicators in suckling goat kid management practices.

Concerning the techniques used to improve meat quality, Abhijith et al. [9] presented a meta-analysis of the effects of electrical stimulation with different voltages and different ageing periods on the meat quality of small ruminants. With this work, it was possible to conclude that electrical stimulation has positive effects in reducing the ultimate pH, Warner–Bratzler shear force, cooking loss, and purge loss. In addition, sarcomere length, the myofibrillar-fragmentation index, and color (L*, a*, b*) showed higher values in meat subjected to electrical stimulation. It was concluded that in having these effects, electrical stimulation is beneficial for the meat quality of small ruminants. On the other hand, Silva et al. [10] presented a review of non-destructive imaging and spectroscopic techniques for assessing carcass and meat quality in sheep and goats. This article reviews the recent technological advances in dual-energy X-ray absorptiometry, computed tomography, and magnetic resonance imaging to assess sheep and goat carcasses and meat quality as non-destructive and non-invasive techniques to provide objective data to evaluate the carcass composition and quality traits of sheep and goat meat. Other optical technologies gaining recognition for monitoring and evaluating the quality and safety of carcasses and meat were also addressed in the review. Attention was paid to visible and infrared reflectance spectroscopy, hyperspectral imagery, and Raman spectroscopy. Practical aspects such as accuracy, reliability, cost, portability, speed, and ease of use were also highlighted for both imaging and spectroscopy techniques.
